# Reaction of Iodonium Ylides of 1,3-Dicarbonyl Compounds with HF Reagents

**DOI:** 10.3390/molecules17066625

**Published:** 2012-05-31

**Authors:** Keisuke Gondo, Tsugio Kitamura

**Affiliations:** Department of Chemistry and Applied Chemistry, Graduate School of Science and Engineering, Saga University, Honjo-machi, Saga 840-8502, Japan

**Keywords:** iodonium ylide, hypervalent iodine, dibenzoylmethane, 1,3-dicarbonyl compound, fluorination, chlorination

## Abstract

Reaction of dibenzoylmethane with (diacetoxyiodo)benzene in the presence of KOH in MeCN quantitatively gave the corresponding iodonium ylide, which was treated with a HF reagent to afford the corresponding 2-fluorinated dibenzoylmethane in 14–50% yields. The similar reaction of the iodonium ylides obtained from 1-phenylbutan-1,3-dione, ethyl benzoylacetate, and ethyl *p*-nitrobenzoylacetate with TEA·3HF gave the corresponding fluorinated products in 17–34% yields. It is suggested that the fluorinated products were formed through the *C*-protonation of the ylide, followed by displacement with fluoride ion. The same reaction of the iodonium ylide of dibenzoylmethane with concentrated HCl gave the corresponding chlorinated product in 45% yield.

## 1. Introduction

**Scheme 1:**
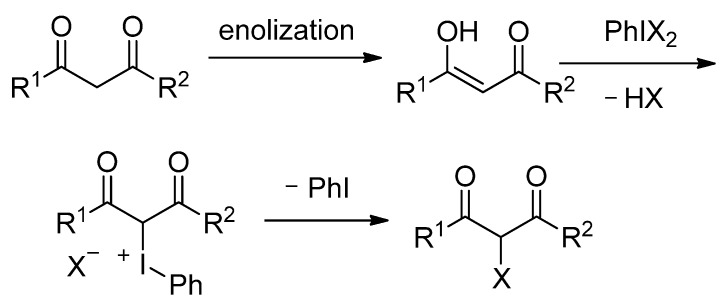
Halogenation of 1,3-dicarbonyl compounds.

Since an iodonium ylide was first prepared by Neilands *et al.* by the reaction of dimedone and (difluoroiodo)benzene [1], many iodonium ylides have been prepared, characterized, and applied to synthetic reactions [2,3,4,5,6,7,8,9]. Most of them are stabilized iodonium ylides that have two strong electron-withdrawing groups such as carbonyl, sulfonyl, cyano, and nitro groups. Unstable monocarbonyl iodonium ylides have been prepared from 2-acetoxyvinyliodonium salts by Ochiai *et al.* [4]. In general iodonium ylides have been used as carbene precursors under thermal, photochemical and catalytic conditions [2,3,4,5,6,7,8,9]. Very recently, we have reported that 1,3-dicarbonyl compounds react with HF or HCl in the presence of iodosylbenzene (PhIO) to give 2-fluoro- or 2-chloro-1,3-dicarbonyl compounds in good to high yields [10,11]. In these reactions, the following mechanism shown in Scheme 1 was postulated. A 1,3-dicarbonyl compound undergoes enolization to form an enol which reacts with (dihaloiodo)benzene to yield the corresponding iodonium halide, followed by displacement with halide anion giving a 2-halogenated 1,3-dicarbonyl compound.

**Scheme 2:**
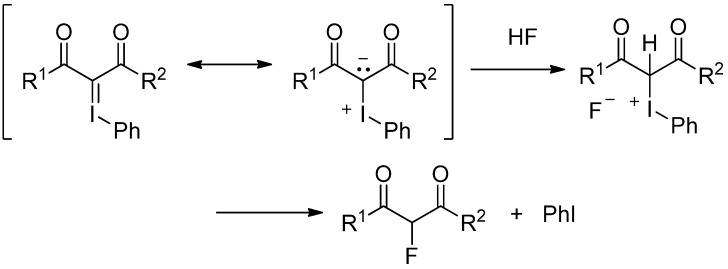
Concept for generation and reaction of iodonium fluorides.

In the above fluorination and chlorination reactions of 1,3-dicarbonyl compounds, the iodonium salts are formed as the key intermediates, which undergo displacement of the phenyliodonio group by halide ion. If the corresponding iodonium halides can be generated by other methods, the same halogenated 1,3-dicarbonyl compounds will be obtained. Although Neilands reported the reaction of the iodonium ylides with protic acids such as HCl, trichloroacetic acid, and *p*-nitrobenzoic acid in methanol [12], until now there are no reports on the reaction of iodonium ylides with HF reagents. Then, we consider a possibility that protonation of the iodonium ylides by HF gives the corresponding iodonium fluorides. The concept is illustrated in Scheme 2. Thus, we have examined the reaction of the iodonium ylides with HF in order to get the information on the possibility of the fluorination reaction of the 1,3-dicarbonyl compounds through the iodonium fluorides.

## 2. Results and Discussion

**Scheme 3:**
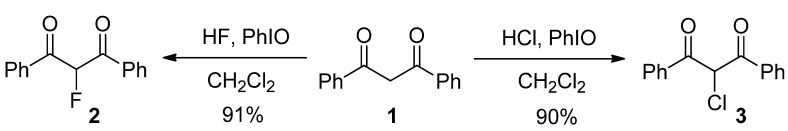
Direct fluorination and chlorination of **1** in the presence of PhIO.

First we chose dibenzoylmethane (**1**) as the 1,3-dicarbonyl compound precursor of the iodonium ylide. Our previous studies indicated that the fluorination and chlorination of **1** in the presence of PhIO give 2-fluoro-1,3-diphenylpropan-1,3-dione (**2**) and 2-chloro-1,3-diphenylpropan-1,3-dione (**3**) in 91 and 90% yields, respectively (Scheme 3) [10,11].

**Scheme 4:**
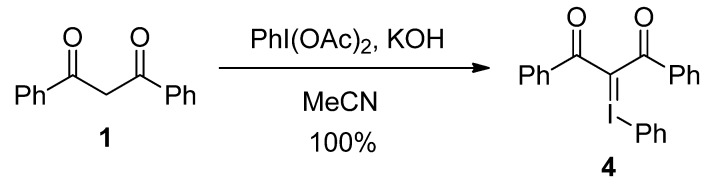
Preparation of iodonium ylide **4**.

As shown in Scheme 4 the iodonium ylide **4** of dibenzoylmethane [12,13] was prepared quantitatively by the reaction of **1** with (diacetoxyiodo)benzene in the presence of KOH in MeCN, according to the recently reported literature method [14]. The iodonium ylide **4** was used directly to the next reaction without further purification due to the instability towards purification processes.

**Scheme 5:**
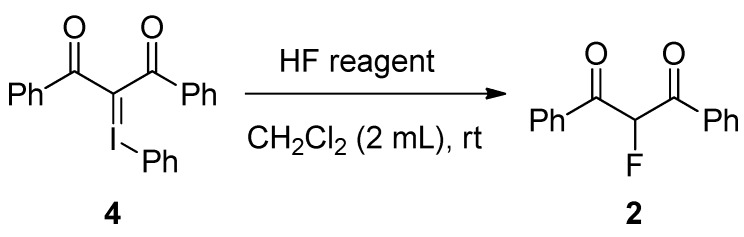
Fluorination of iodonium ylide **4**.

Next, with the iodonium ylide of dibenzoylmethane in hand, we examined the reaction of **4** with HF under several conditions (Scheme 5). The results are given in Table 1. Treatment of the iodonium ylide **4** with 55% hydrofluoric acid (five equiv of HF) in CH_2_Cl_2_ at room temperature for 17 h afforded 2-fluoro-1,3-diphenylpropan-1,3-dione (**2**) in 20% yield (Entry 1). Since we considered that the lower yield of fluorinated product **2** might be attributable to a lower nucleophilicity of fluoride ion by hydration of water, we used triethylamine complexes with HF (TEA·nHF) instead of aqueous hydrofluoric acid. Using TEA·3HF complex as the HF reagent much improved the yield to give the fluorinated product **2** in 45% yield (Entry 2). We then examined the reaction with TEA·5HF complex having a higher content of HF. The reaction of **4** with TEA·5HF complex under the similar conditions gave the fluorinated product **2** in 32% yield (Entry 3). The reaction of **4** with TEA·3HF complex for 1.5 h afforded a similar result to Entry 3 (Entry 4). Finally, we examined the inverse addition, that is, a solution of **4** in CH_2_Cl_2_ was added to a solution of TEA·3HF in CH_2_Cl_2_. The result was somewhat improved to give the fluorinated product **2** in 50% yield (Entry 5). To compare with the direct fluorination of **1** with HF/PhIO, the reaction was conducted under the same conditions (40 °C, 36 h) (Entry 6). However, the yield of **2** was decreased to 14%. In all experiments, an inseparable complex mixture was observed in this reaction.

**Table 1:** Reaction of iodonium ylide **4** with HF reagents ^a^.

Entry	HF reagent	Time (h)	Yield of 2 (%) ^b^
1	55% HF	17	20
2	TEA·3HF	17	45
3	TEA·5HF	17	32
4	TEA·3HF	1.5	41
5	TEA·3HF ^c^	1.5	50
6	TEA·3HF ^c,d^	36	14

^a^ Conditions: **4** (1 mmol), a HF reagent (5 mmol), CH_2_Cl_2_ (2 mL), rt; ^b^ Isolated yield by column chromatography on silica gel; ^c^ Inverse addition, CH_2_Cl_2_ (70 mL); ^d^ At 40 °C.

**Scheme 6:**
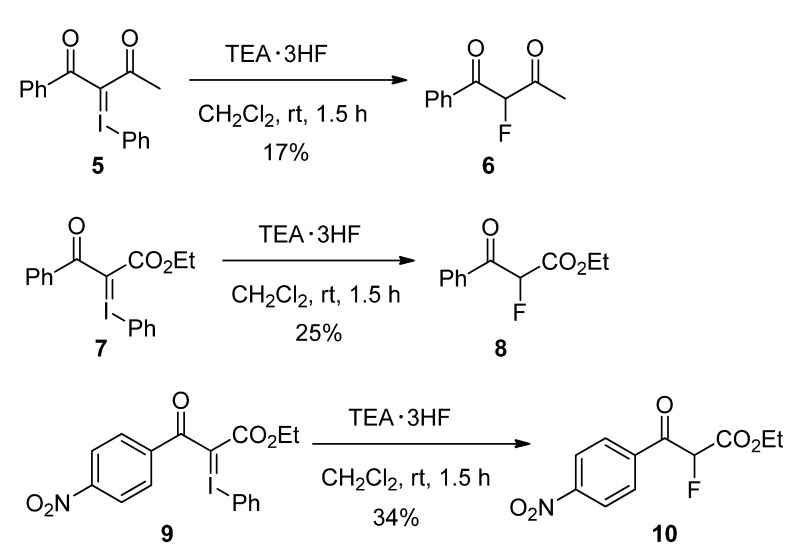
Scope of fluorination of iodonium ylides.

We further examined the fluorination reaction with TEA·3HF complex, concerning with other iodonium ylides, as shown in Scheme 6. The reaction of iodonium ylide **5** prepared from 1-phenylbutan-1,3-dione [15] gave the fluorinated product **6** in 17% yield. The iodonium ylides of β-keto esters also showed the similar results. The reaction of iodonium ylide **7** prepared from ethyl benzoylacetate [15] afforded the fluorinated product **8** in 25% yield, and the iodonium ylide **9** of ethyl *p*-nitrobenzoylacetate provided the fluorinated product **10** in 34% yield. The above results indicate that the fluorination of the iodonium ylides takes place, but the yields of fluorinated products are poor to moderate.

**Scheme 7:**
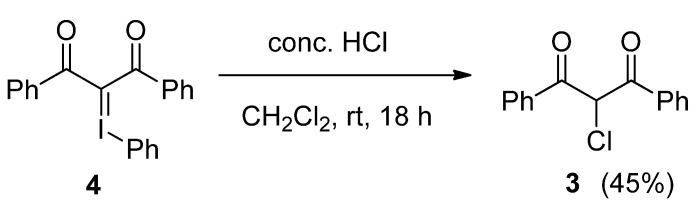
Chlorination of iodonium ylide **4**.

Although we sought to study the above reaction with HF, we also examined about the reaction with hydrochloric acid at this time. When the iodonium ylide **4** was treated with concentrated hydrochloric acid in CH_2_Cl_2_ at room temperature for 18 h, 2-chloro-1,3-diphenylpropan-1,3-dione (**3**) in 45% yield (Scheme 7). This result is similar to that of the fluorination of **4**.

**Scheme 8:**
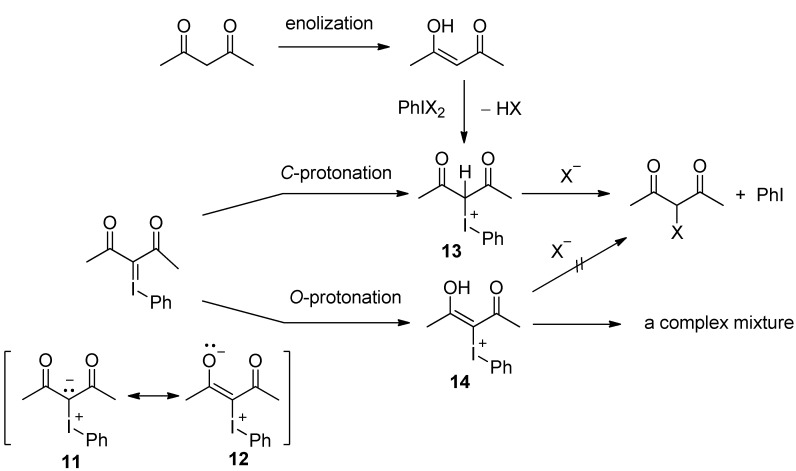
A possible mechanism.

From the above results, it became clear that the fluorination or chlorination of iodonium ylides with HF or HCl gives the corresponding halogenated products. However, the yield of the halogenated products is lower than that obtained by the reaction of 1,3-dicarbonyl compounds with HF or HCl in the presence of PhIO [10,11]. As shown in Scheme 8, we considered the following: in the reaction of an iodonium ylide with HX, protonation occurs first. Since the iodonium ylide has resonance structures such as **11** and **12**, two types of protonation may exist in the reaction of the iodonium ylide with HX. The *C*-protonation gives desired iodonium halide **13**, which leads to the formation of a halogenated product by displacement. On the other hand, the *O*-protonation leads to the formation of vinyliodonium halide **14**. Accordingly, the *O*-protonation path does not give the desired halogenated product but causes to decomposition to a complex mixture because the vinyliodonium salts formed by *O*-protonation of the ylide do not undergo the substitution reactions under the present mild conditions.

## 3. Experimental

### 3.1. General

*Caution: Hydrofluoric acid (HF) in contact with the skin causes very painful burns. Exposed parts of the body must be protected when handling it.* All solvents and starting materials were used as received without further purification unless otherwise indicated. ^1^H-NMR (300 MHz), ^13^C-NMR (75 MHz), and ^19^F-NMR (282 MHz) spectra were recorded on a JEOL JNM-Al 300 FT-NMR spectrometer (Tokyo, Japan) in CDCl_3_ solution (TMS as an internal standard). Melting points were measured with a YANACO micro melting apparatus and are uncorrected. Column chromatographic separations were carried out using silica gel as the stationary phase. Pre-coated plates (silica gel 60 F_254_, MERCK, Damstadt, Germany) were used for TLC examination.

### 3.2. General Procedure for the Reaction of Iodonium Ylides with HF or HCl Reagents

The preparation of the iodonium ylides was conducted according to the literature method [14]. Under argon atmosphere, a mixture of KOH (6 mmol), a 1,3-dicarbonyl compound (1 mmol), and MeCN (5 mL) was cooled at 0 °C (ice/water bath) and stirred vigorously for 5 min. PhI(OAc)_2_ (1.1 mmol) was then added and the reaction mixture was stirred vigorously at 0 °C for 20 min. Water (30 mL) was added, and the mixture was stirred for 1 min. The resulting solid was filtered, washed with water (2 × 2 mL). The solid was finally washed with ether (2 mL) and then directly used for fluorination reaction. The iodonium ylide prepared above was dissolved in CH_2_Cl_2_ (70 mL). The solution of the iodonium ylide in CH_2_Cl_2_ was added dropwise to a stirred solution of TEA∙3HF (5 mmol) in a Teflon tube and the reaction mixture was stirred at room temperature for 1.5 h. The reaction mixture was neutralized with aqueous NaHCO_3_ and extracted with CH_2_Cl_2_ (3 × 5 mL). The combined organic extract was washed with brine, dried over anhydrous Na_2_SO_4_, and concentrated under a reduced pressure. The fluorinated product was separated by column chromatography on silica gel (hexane/EtOAc).

*2-Fluoro-1*,*3-diphenyl-1*,*3-propanedione* (**2**) [16]. M.p. 66.5–67.7 °C (hexane); ^1^H-NMR (CDCl_3_) δ 6.54 (d, *J* = 49 Hz, 1H, CH), 7.43–7.50 (m, 4H, ArH), 7.56–7.63 (m, 2H, ArH), 8.07-8.10 (m, 4H, ArH); ^13^C-NMR (CDCl_3_) δ 96.48 (d, *J* = 197.2 Hz), 128.72, 129.74 (d, *J* = 3.1 Hz), 133.48 (d, *J* = 1.8 Hz), 134.45, 191.12 (d, *J* = 19.8 Hz); ^19^F-NMR (CDCl_3_) δ −188.20 (d, *J* = 49 Hz).

*2-Fluoro-1-phenyl-1*,*3-butanedione* (**6**) [16]. ^1^H-NMR (CDCl_3_) δ 2.34 (d, *J* = 4.2 Hz, 3H, Me), 5.95 (d, *J* = 50 Hz, 1H, CH), 7.46–8.03 (m, 5H, ArH); ^13^C-NMR (CDCl_3_) δ 25.92, 96.51 (d, *J* = 197.2 Hz), 128.70, 129.64 (d, *J* = 3.1 Hz), 133.41, 134.56, 190.23 (d, *J* = 19.1 Hz), 200.49 (d, *J* = 23.5 Hz); ^19^F-NMR (CDCl_3_) δ −182.54 (dq, *J* = 4.2, 50 Hz).

*Ethyl 2-fluoro-3-oxo-3-phenylpropionate* (**8**) [17]. ^1^H-NMR (CDCl_3_) δ 1.26 (t, *J* = 7.2 Hz, 3H, Me), 4.30 (q, *J* = 7.2 Hz, 2H, CH_2_), 5.87 (d, *J* = 48 Hz, 1H, CH), 7.48–7.53 (m, 2H, ArH), 7.61–7.67 (m, 1H, ArH), 8.03–8.06 (m, 2H, ArH); ^13^C-NMR (CDCl_3_) δ 13.84, 62.59, 89.95 (d, *J* = 196.0 Hz), 128.75, 129.43 (d, *J* = 3.8 Hz), 133.36 (d, *J* = 1.9 Hz), 134.43, 164.83 (d, *J* = 24.2 Hz), 189.47 (d, *J* = 19.7 Hz); ^19^F-NMR (CDCl_3_) δ −190.57 (d, *J* = 48 Hz).

*Ethyl 2-fluoro-3-(4-nitrophenyl)-3-oxopropionate* (**10**) [18]. ^1^H-NMR (CDCl_3_) δ 1.29 (t, *J* = 7.2 Hz, 3H, Me), 4.34 (dq, *J* = 1.5, 7.2 Hz, 2H, CH_2_), 5.95 (d, *J* = 49 Hz, 1H, CH), 8.22–8.38 (m, 4H, ArH); ^13^C-NMR (CDCl_3_) δ 13.80, 63.03, 90.08 (d, *J* = 197.2 Hz), 123.79, 130.55 (d, *J* = 4.4 Hz), 137.59 (d, *J* = 2.5 Hz), 150.76, 164.06 (d, *J* = 23.5 Hz), 188.51 (d, *J* = 21.0 Hz); ^19^F-NMR (CDCl_3_) δ −191.81 (d, *J* = 49 Hz).

*2-Chloro-1*,*3-diphenylpropan-1*,*3-dione* (**3**) [19]. M.p. 86−87 °C; ^1^H-NMR (CDCl_3_) δ 5.30 (s, 1H, CH), 7.11 (t, *J* = 7.2 Hz, 4H, ArH), 7.33 (t, *J* = 7.2 Hz, 2H, ArH), 7.71 (d, *J* = 7.2 Hz, 4H, ArH); ^13^C-NMR (CDCl_3_) δ 62.14, 128.79, 128.97, 133.64, 134.15, 189.28.

## 4. Conclusions

In conclusion, we have demonstrated that the reaction of iodonium ylides of 1,3-dicarbonyl compounds with HF provides the corresponding fluorinated product derived from the *C*-protonation of the ylides, followed by displacement with fluoride ion, although the yield of the fluorinated product is not high compared with that of the reaction of 1,3-dicarbonyl compounds with HF in the presence of PhIO. The chlorination reaction also proceeds similarly. Thus, it is postulated that there exists an *O*-protonation of the ylides forming vinyliodonium salts.
